# Feasibility study exploring sleep impacts among families in the aftermath of firearm-related trauma

**DOI:** 10.3389/frsle.2026.1787037

**Published:** 2026-08-04

**Authors:** Rebecca Robbins, Peter T. Masiakos, Grace Vlaun, Stuart F. Quan, Chana A. Sacks

**Affiliations:** 1 Harvard Medical School, Boston, MA, United States; 2 Division of Sleep and Circadian Medicine, Department of Medicine, Brigham and Women's Hospital, Boston, MA, United States; 3 Gun Violence Prevention Center, Massachusetts General Hospital, Boston, MA, United States; 4 Department of Surgery, Massachusetts General Hospital, Boston, MA, United States; 5 Harvard College, Cambridge, MA, United States; 6 Division of General Internal Medicine, Massachusetts General Hospital, Boston, MA, United States

**Keywords:** caregiver, gun violence, mindfulness, post-traumatic stress disorder, sleep, sleep difficulties

## Abstract

**Objective:**

The ripple effects of gun violence are far reaching, particularly for children impacted by this trauma. Many of the available sources of mental health support in the wake of gun violence do not address disrupted sleep, which commonly develops as a consequence of loss.

**Method:**

We recruited adults for focus groups who are caregivers to a child or young adult impacted by loss due to gun violence from the Victim Support Services department of a police department in the US Southeast. The children to whom the adult provides care were required to be 13 years of age or younger. The aim of the focus groups was to understand sleep difficulties and ascertain the feasibility of a mindfulness-based behavioral intervention smartphone application (app) for sleep. Interviews were audio recorded, transcribed verbatim, and then analyzed in accordance with the constant comparison method.

**Results:**

The average age of caregivers in the sample (*n* = 8) was 50 years of age (s.d. = 8.9); 100% were female and 100% were Black/African American. Themes from the qualitative analysis suggested that caregivers experienced sleep difficulties that were intimately related to grief and trauma due to loss. Caregivers also described sleep difficulties in their children in the wake of loss. Participants provided favorable reactions to the mindfulness app as feasible and appropriate for families like them.

**Conclusions:**

Focus groups revealed sleep difficulties among families impacted by loss due to gun violence. Reactions to the mindfulness app were positive. Future research should evaluate the impact of the mindfulness intervention on child and caregiver mental and sleep health.

## Introduction

The number of firearm-related deaths in the US is staggering (>48,000 in 2022) (Gun Violence Archive, 2022; WISQARS Explore Fatal and Nonfatal Data, n.d.), but beyond those killed by firearms, gun violence leaves behind parents, spouses, and children, who suffer profound grief in the wake of their loss. Involvement in violent acts or exposure to violence in youth is associated with short- and long-term health impacts. Youth who report being in a fight or being the victim of bullying also report more loneliness and more insomnia than those without these experiences (Sharma et al., 2017). Youth who report exposure to violence (e.g., witnessing a homicide) demonstrate worse mental and cardiovascular health than those without such exposures (Sheats et al., 2018). Less attention has been paid to sleep-related interruptions due to gun violence exposure, such as those who may have witnessed a loved one be shot. Similarly, there is a insufficient concern toward those who are suffering from the traumatic ripple effect of the loss of a loved one in a violent manner, even though the nighttime burden of firearm injuries is substantial (Klerman et al., 2023; Robbins et al., 2024).

Previous research has explored the design of behavioral interventions aimed at supporting youth with violence exposure. Interventions have been developed to support persons exposed to frequent gun violence. For instance, research has equipped providers that support youth exposed to violence with trauma-informed counseling skills (Damian et al., 2019). Research has also found that programs offering paid, after-school employment among youth exposed to violence can improve mental health symptoms, such as feelings of anxiety (Cromer et al., 2019).

According to a meta-analysis, mindfulness-based interventions are effective for improving feelings of stress, anxiety, and depression in youth (Kallapiran et al., 2015). Given the role of mindfulness in supporting stress management and mental health among youth, and that that sleep difficulties typically co-occur with mental health concerns among those exposed to violence (Gibson et al., 2019; Lavie, 2001; Sandahl et al., 2017), a mindfulness-based intervention holds promise as a tool for supporting youth and families impacted by violence.

Thus, we conducted a feasibility study of a mindfulness-based intervention to support sleep among children and caregivers impacted by violence. Specifically, we first explored sleep difficulties and experience with mindfulness among children and caregivers impacted by violence. Then, we delivered a brief mindfulness-based intervention and collected reactions from caregivers.

## Methods

We conducted focus groups with family members of victims of firearm violence who are currently caring for children. The aims of the focus groups were to understand sleep and opportunities to support families, including caregivers and children, who have been impacted by firearm violence and collect preliminary feedback to a mindfulness-focused behavioral intervention for children impacted by loss due to firearm violence (e.g., loss of a mother, father, or other family member) and their caregivers. This study was approved by the IRB at Mass General Brigham (Boston, MA).

### Participants and recruitment

We collaborated with stakeholders, Victim Support Specialists at a police department in a mid-sized US city in the Southeast, to identify individuals who are family members of victims of firearm violence. Eligible participants also reported caring for one or more children aged 13 or younger who were also impacted by the violence exposure. Participants were recruited through flyers and emails from the Victim Support personnel. Once we had enough participants for a focus group, we scheduled a Zoom call. The Zoom calls were audio recorded and transcribed verbatim. During each focus group, 6 participants were recruited and 4 attended.

### Focus groups

Two focus groups were conducted. Each focus group included 4 participants. The focus groups were conducted by the senior author (RR), an experienced female qualitative researcher and Assistant Professor who completed 2 graduate-level qualitative research classes and has undertaken >15 qualitative research projects. Focus groups lasted approximately 90 minutes.

### The mindfulness-based behavioral intervention

**Figure 1:**
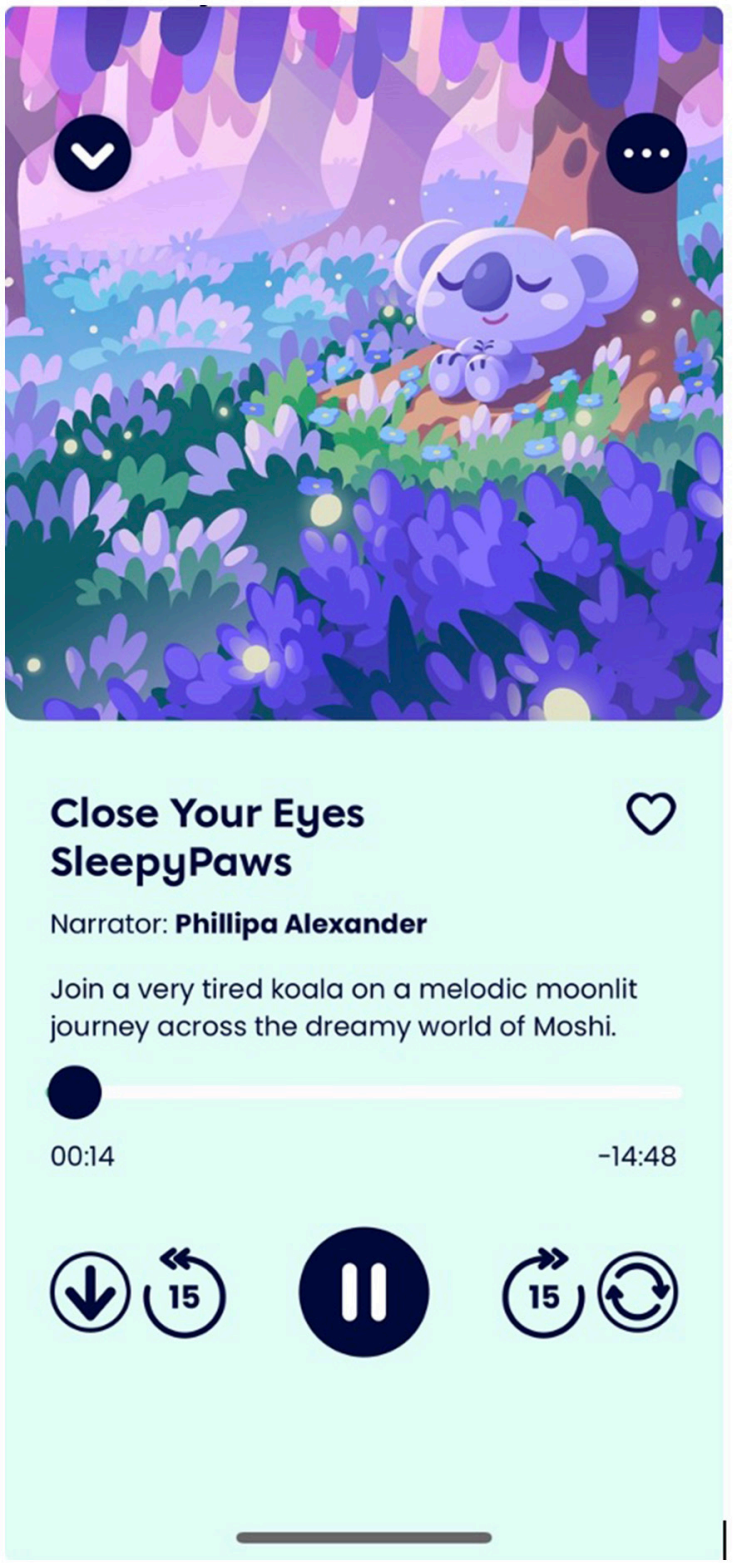
Screenshot of the smartphone-based, story-focused intervention delivering mindfulness through stories and songs featuring several stories. Participants listened to 2 stories during the present focus group study and provided open-ended feedback after listening to the story.

In this study, we explored the feasibility of a mindfulness-based sleep improvement behavioral intervention for children and early adolescents. The intervention is a mindfulness smartphone application (app) designed to deliver mindfulness principles (e.g., visualization, breathing exercises) through a story (see Figure 1). The app allows users to select a story of their choosing, then invites users to put their phone down and listen to the story with their eyes closes to imagine the characters and events. The app has been previously tested and shown to improve sleep health habits in children (age 3–8 years of age) (Chung et al., 2022). In the present study, we explored the feasibility of using this intervention in children, adolescents, and adults impacted by firearm violence. Moshi, the creators of the App, provided free access for the purpose of these focus groups. The company otherwise had no role in the study, including in data collection and analysis or the decision to submit for publication.

### Interview guide and study procedure

The first draft of the interview guide for the focus groups was developed by the First and Senior Author. All members of the research team provided substantial intellectual input to the interview guide. Finally, a meeting was scheduled with our Victim Support Specialist colleagues to discuss the interview guide and solicit their feedback. The Victim Support Specialists provided important feedback. For instance, an earlier version of the interview guide asked questions about the death of their loved ones, and the relationships among the participants, victim, and children. Our Victim Support Specialists provided important feedback on the potential negative consequences of the triggering nature of these questions. The interview guide then was edited to focused solely on sleep among child and caregivers, and reactions to the mindfulness-based behavioral intervention.

The final version of the interview guide (provided in the Appendix) was a semi-structured guide that began with the interviewer describing the research goals. Then, the interviewer asked a series of open-ended questions about sleep, such as “Can you tell me about your sleep in the wake of your loss?” Next, caregivers were questioned about their child's sleep. Then, participants were asked about their experience with mindfulness. Finally, participants watched a story from the Moshi story-based mindfulness app. The app features a library of stories with relaxing sounds, stories, and music. Users pick a story, put down their phone or other device and listen and practice the breathing exercises. In the focus groups, the leader played one of the stories from the Moshi app and did a mindfulness exercise. After these two activities were over, participants were asked for their reactions with questions such as “What did you like about the activity?” and “What did you *not* like about the activity?” In addition, the interviewer took notes during the interview process noting important observations, emergent themes, and other details.

### Analysis

The transcripts were recorded and transcribed verbatim. Next, transcripts were cleaned to remove interviewer questions or discussion that was extraneous to the study aims. There were no ambiguities identified in the transcripts, so they were not returned to participants for clarification. Once the transcripts were cleaned, coding commenced. The coding procedure was conducted under the supervision of the senior author who is an experienced qualitative researcher (RR) and a Bachelor-level trained and experienced Research Assistant (GV). Coding proceeded in accordance with the constant comparative method through several iterative stages (Charmaz, 2006). Coding was done in Microsoft Excel. First, coders conducted a first reading whereby they oriented themselves to the data without assigning codes. Second, the coders were instructed to re-read the transcripts and iteratively assign codes to each utterance. Then, the coders met to conduct a line-by-line review of each other's codes and discuss any discrepancies between the coders until they reached agreement on a revised list of codes. Then, coders re-coded each transcript with the new, agreed-upon codes. In each round of coding and discussion, the authors achieved a smaller, more concise number of overall codes. There were two rounds of coding, discussion, and re-coding. After the second round, in their meeting to review each other's line-by-line codes, the two coders reached agreement on their codes and coding was deemed complete.

## Results

All participants (*n* = 8) were female (100%) and Black/African American (100%). The mean (SD) age of participants was 50.4 (8.9) years. Participants reported caring for 2.7 children on average. The mean age of the children for whom participants provided care was 11.1 years.

### Profound sleep difficulties among caregivers impacted by firearm violence

Participants shared their experiences with the profound grief associated with losing a loved one. As one participant shared:

“I mean when I go around them, we don't talk about her because they‘re not ready for that yet, so I understand it's deeper than you know like I told people, it's deeper than a heart pain, it's to your soul, it really is, yes, and I wish that whatever study you're doing in in regards to children and trauma you know, my grandson. he watched me [cry] like I was a baby.”

In addition to the profound grief associated with loss of a loved one, several participants shared frustration with injustices associated with their loss. As one participant described:

“Besides the murder, I just continued to see the injustice that was given to us when we went to trial.”

The experience of profound sleep difficulties was a consistent theme throughout the focus groups with caregivers. Among the sleep difficulties, one issue voiced by many participants was an inability to fall asleep due to recurring thoughts about the loss of their loved one. As shared by one participant concerning the inability to fall asleep due to persistent thoughts about their loss:

“But, on the other hand myself, it's like, huh! I don't. I don't sleep. because it feel like when I close my eyes. All I do is, you know, is think about what happened, because my son was actually murdered in front of me.”

One common experience participants shared was anxiety about falling asleep. Several participants reported these anxieties stemmed from their loss, such as fear of falling asleep and missing an important call or losing another loved one. The following quote captures this poignantly:

“You know I wanted to be up. I didn't want to go to sleep, and I think your brain kind of goes into that trauma mode, but also that mamma mode. If I sleep, I'm going to miss something, you know. Because I was waiting on the detectives to call me, give me some answers, you know. I didn't have a lot of people that I wanted to talk to at that time.”

Several participants reported reliance upon pharmacological sleep aids, over-the-counter medications, and alcohol. One quote from a participant captures the profound difficulties relating to falling asleep, and the use of a pharmacological sleep intervention:

“Sleep was something that was really hard to come by. Just couldn't seem to, I guess your mind just can't seem to quite rest, you know, or get to a place where you have some peace and can relax. So for me, I did go to my doctor after some time and tried some sleep aids or tried a sleep aid for a time had something that was supposed to help me sleep, and then something that was supposed to bring the anxiety down. So I was on a couple of medications for a while.”

Another element of the sleep difficulties participants reported in the wake of their loss were nightmares. One quote from a participant captures this:

“I guess it's probably a question for my therapist, but I have had a few nightmares. I've never had nightmares before, and I'm one of the people that look into meanings of things and like, well, maybe my mom's trying to tell me something or whatever. But it's the same one kind of over and over. So do you suggest what I have those to wake up and try to meditate? Or do I write them down? If how do I?”

Finally, the last element of the overarching theme relating to sleep difficulties was oversleeping. Participants described fears of waking up to face the reality of their loved one being lost. One participant quote captures these fears:

“And so as far as sleeping, I sleep too much, and I think because I don't want to wake up face to day without him. I it's hard to go, you know you're waking up, and here it is all over again. Another day of pain another day without your child and you have to go through that all a whole 24 h the reality of you don't get to see or talk to your child again face with all the memories, face with all the pain phase, with all the anger that you had, you know, with the person who did it. I I'd rather say sleep, and that's where it comes, and you know some people wish that they wish they wouldn't even hear on Earth cause it's so painful.”

### Sleep difficulties and nightmares among children and youth impacted by gun violence

Approximately half of the caregivers shared sleep difficulties that their child experienced after their loss. Caregivers reported the difficulties in some cases stemmed from being exposed directly to the violent act that resulted in the death of a loved one:

“As it relates to my grandson, he was 18 months old when his father died, and he actually witnessed his father's murder in the apartment. He witnessed the the effects of his father being shot and falling. And you know all of that happening at 18 months old. And so you know of course, we have paid a lot of attention to him, just to see what the effects of that may or may not be, you know, wanting to be on top of it. That was going to have some, you know, effect on him, and his sleeping has been affected, you know.”

Similarly, another participant described their daughter's sleep difficulties being linked to having witnessed her brother being murdered. The participant also shared techniques the participant used with daughter, such as playing calming music, for her daughter:

“But my daughter, she's 21, and she is still going through because she also witnessed her brother being murdered. So it's like we have to, you know call me at 10 o'clock when she can't sleep, and just me being on that other phone up at the end of the phone, she's able to go to sleep because now she's, you know, at peace, just because Mom is on the other end, and I'm setting up, you know, with her. Either I'm singing or I'm playing waterfalls. I'm playing music for her, would be able to find that piece to go to sleep and that's what I think we need you know, as well.”

The above quote also highlights how these sleep difficulties, due to grief and the trauma associated with observing a loved one be killed, can wane with time. Another aspect of the difficulties caregivers reported had to do with nightmares. One participant quote captures their child's experience of nightmares:

“He has nightmares. He would you know say to me that, grandma don't want to go to sleep, you know. Just you know I'm gonna have nightmares if I go to sleep. So I know that it. That situation affected him, you know, or at least I attribute what he saw, you know. And now, being older and thinking about it, you know, has impacted his ability to be able to have a good night's sleep… You know, even at that age, he has held that in his psyche to some degree, and is impacted, you know, by that. So he has had some issues. With sleep as well, just restless, and then just not even wanting to go to sleep. And then having nightmares, you know”

### Experience with meditation and mindfulness

Across the two focus groups, only three participants reported having practiced mindfulness. All the participants who mentioned practicing mindfulness reported that a therapist had suggested they try mindfulness:

“Yeah, and I'm in therapy. So my therapist recommends I do those, we do them in my sessions as well.”

Another participant reported doing a body scan, a specific mindfulness practice, that they learned with their therapist, and it being helpful for their anxiety:

“Yeah, I do that, and I also do something called a body scan. And it, yeah, it helps the body scan helps more than the meditation does for me. But yeah, I do them occasionally. When I have high anxiety, it helps.”

In this quote, a participant shares that they enjoy the practice of mindfulness, but that being in silence can be difficult for them, and that they prefer to ‘stay busy' to keep their mind off of their loss:

“Sometimes I don't like the silence right now. As it's kind of getting to that space where I could be still. I overwork myself so I don't have to feel right now. So hopefully I can change that.”

### Reactions to the mindfulness intervention

Participants shared overall positive evaluations of the tool, including liking the narrator and calming music:

“I like the calm voice, and of course, the music. I like music.”

Another participant mentioned liking the on-demand nature of the program and the ability to replay as needed:

“Yeah. I like the on-demand and ability to replay, if you need it a little more.”

Another participant commented on the visualizations in the tool:

“I like the visualization of you know, putting a visual in your mind, closing your eyes. Imagine where you going and all that that was really detailed in the vision of, you know, creating an experience of meditating. But I do like that one. Probably one of my favorites.”

The below comment captures an observation shared by several, which is that the mindfulness intervention was appropriate for families who have experienced loss, such as due to firearm violence:

“I think that you know, as a family experiences this loss, you know, and navigates through it together. If there is a mom or other family member who is listening to this meditation with one of their younger children, I think certainly, that there will be some benefit to whoever is listening. You know, even the older you know, parent or guardian, because it certainly has helped to excuse me, regulate my emotions, you know a bit as I was listening to. So thank you for sharing that.”

One participant commented that the music sounded like gospel music, which she found comforting:

“And when the music first came on, it sounded like a gospel song. And it almost maybe say, you know, I want to listen to some gospel music after I am done with this this meeting. I liked it. I really enjoy it.”

Additional quotes can be found in the Table 1.

**Table 1:** Qualitative themes summarizing caregiver sleep difficulties, child sleep difficulties, and reactions to the mindfulness tool.

Theme	Code	Sample quote
Caregiver sleep difficulties		
Sleep difficulties	Inability to sleep	But, on the other hand myself, it's like, huh! I don't. I don't sleep. because it feel like when I close my eyes. All I do is, you know, is think about what happened, because my son was actually murdered in front of me.
	Anxiety about falling asleep	You know I wanted to be up. I didn't want to go to sleep, and I think your brain kind of goes into that trauma mode, but also that mamma mode. If I sleep, I'm going to miss something, you know. Because I was waiting on the detectives to call me, give me some answers, you know. I didn't have a lot of people that I wanted to talk to at that time.
	Oversleeping	And so as far as sleeping, I sleep too much, and I think because I don't want to wake up face to day without him. I it's hard to, you know you're waking up, and here it is all over again.
	Nightmares	At first I had a hard time sleeping. I would see lightning flashes. I would see these lightning flashes when I slept next alone is loud bang in my hand. Banging noise of my head, and just see lightning flashes. And so I did look it up, and it's a actual disorder at the trauma. I can't recall what the name of it is now.
Sleep aids	Use of alcohol	My sister helped me through it. I did resort to some drinking to help me to sleep.
	Use of sleeping pills	Sleep was something that was really hard to come by. Just couldn't seem to, I guess your mind just can't seem to quite rest, you know, or get to a place where you have some peace and and can relax. So for me, I did go to my doctor after some time and tried some sleep aids or tried a sleep aid for a time had something that was supposed to help me sleep, and then something that was supposed to bring the anxiety down. So I was on a couple of medications for a while.
	Use of over-the-counter sleep aids	For sleep, I take Advil PM every night, which I'm afraid that's gonna mess with my kidneys later on down the line. I don't sleep through the night. I constantly check on my son throughout the night. Just a lot of, you know I just put a ring camera on my door, so I'm just kind of always scared or nervous. I don't know
Caregiver-reported child sleep difficulties		
Sleep difficulties	Trauma associated with gun violence exposure	But my daughter, she's 21, and she is still going through because she also witnessed her brother being murdered. So it's like we have to, you know call me at 10′clock or 20′clock when she can't sleep, and just me being on that other phone up at the end of the phone, she's able to go to sleep because now she's, you know, at peace, just because Mom is on the other end, and I'm setting up, you know, with her. Either I'm singing or I'm playing waterfalls. I'm playing music for her, would be able to find that piece to go to sleep. And that's what I think we need. You know as well.
	General difficulty	And as far as my children, they can't sleep.
Nightmares	Fear of sleeping due to nightmares	He has nightmares. Noah would you know say to me that, grandma don't want to go to sleep, you know. Just you know I'm gonna have nightmares if I go to sleep. So I know that it. That situation affected him, you know, or at least I attribute what he saw, you know. And now, being older and thinking about it, you know, has impacted his ability to be able to have a good night's sleep. Well, no, we‘re gonna say our prayers, will, mom? Well, grandma, what if Jesus, I say, you know, if you're getting a nightmare. Just say, Jesus, Jesus will, grandma, what if Jesus won't save me? You know. So just yeahso it is my belief that what he experienced. You know, even at that age, he has held that in his psyche to some degree, and is impacted, you know, by by that. So he he has had some issues. With sleep as well, just restless, and then just not even wanting to go to sleep. And then having nightmares, you know
Reactions to the mindfulness tool		
General positive evaluations	Liked the narrator	I like the calm voice, and of course, the music. I like music.
	Liked the music	The calm voice, the music is really great.
	Liked on-demand nature	Yeah. I like the on-demand and ability to replay, if you need it a little more
	Liked visualization	I like the visualization of you know, putting a visual in your mind, closing your eyes. Imagine where you going and all that that was really detailed in the vision of, you know, creating an experience of meditating. But I do like that one. Probably one of my favorites.
Appropriate for families	Caregivers and child to benefit	I think that you know, as a family experiences this loss, you know, and navigates through it together. If there is a mom or other family member who is listening to this meditation with one of their younger children, I think certainly, that there will be some benefit to whoever is listening. You know, even the older you know, parent or guardian, because it certainly has helped to excuse me, regulate my emotions, you know a bit as I was listening to. So thank you for sharing that.
	Potential for older children (teens) to benefit	It would be great if there is something because, even as adults, if we feel a benefit from just hearing these meditations. And I have a strong feeling that our teens could benefit too from this calm, relaxing music to help bring down their blood pressure, help them relax
Culturally appropriate	Music sounded like gospel	And when the music first came on, it sounded like a gospel song. And it almost maybe say, you know, I want to listen to some gospel music after I am done with this this meeting. I liked it. I really enjoy it.

## Discussion

We explored the experience of sleep among families who have endured loss due to firearm violence and captured preliminary reactions to a potential intervention for supporting sleep and mindfulness in families impacted by firearm violence. In the focus group discussions, all caregivers reported sleep difficulties in the wake of their loss. Participants described trauma and anxiety being intimately related to their sleep difficulties. For instance, several participants reported dreading the nighttime when their trauma would resurface. Others reported inability to fall asleep due to fears of missing a call or the opportunity to receive information about the person they lost. Participants, in several instances, also described their children's sleep difficulties and nightmares, but in less detail than they described their own. This lack of detail could suggest either that their children were either not experiencing sleep difficulties with as much prevalence as the caregiver, or that children were not expressing their difficulties. In either case, these focus groups did reveal profound sleep difficulties among caregivers, which could lend further support for the present approach which aims to support children and youth *and* their caregivers with an intervention intended to be experienced in the context of a child-caregiver dyad. Future research may consider engaging members of this population to appropriately tailor and culturally adapt the mindfulness intervention prior to implementation.

Experiences with mindfulness were reported by less than half of participants, but of those who reported experience with mindfulness, several reported practicing sound therapy with a therapist. Participants provided positive evaluations of the mindfulness app that was piloted in this study. Participants indicated that the tool would be appropriate and feasible for families, such as themselves, and that caregiver and child stand to benefit from the intervention. One participant reported that the music sounded like gospel, which she found to be comforting and culturally appropriate. The qualitative insights and initial experiences with the mediation tool introduced here set the stage for further research to evaluate this intervention to understand the impact of the mindfulness tool on the sleep and mental health of caregivers and children experiencing loss due to firearm violence.

Previous research has evaluated programs aimed at supporting youth impacted by violence, such as trauma-informed counseling skills training for providers supporting youth (Damian et al., 2019), or providing youth with after school job opportunities (Cromer et al., 2019). According to a review of evidence-based approaches for supporting youth with adverse child experiences that include but are not limited to violent exposure, mindfulness is among the most effective strategies for supporting these young people (Gilgoff et al., 2020). Despite this, few programs have specifically explored sleep or mindfulness-focused interventions among youth impacted by violence, such as loss of a loved one due to firearm violence. Given the previous evidence to show that violent exposure is associated with sleep and mental health concerns (Gibson et al., 2019; Lavie, 2001; Sandahl et al., 2017), such programs are warranted. Further, the mindfulness intervention proposed herein intervenes at the level of the child-caregiver dyad, which may extend the impact of the program beyond youth themselves to other members of the family unit. Future research evaluating the impact of the proposed intervention is necessary to ensure it is acceptable and appropriate for improving sleep and mental health among children/youth and families impacted by firearm violence.

While not a representative sample, the preponderance of Black/African American individuals who volunteered for the study highlights the disparities in violence exposure. Unfortunately, exposure to community violence (e.g., witnessing an assault) among children in the US is far too high (i.e., reported by 25% of children) (Finkelhor et al., 2009). Moreover, violent exposure and violent victimization disproportionately affects children of color (Umlauf et al., 2011). Given the greater burden of violent exposure and violent victimization, it is vital that interventions are designed to support these communities in the wake of violence.

Limitations of this research include the small sample and thus it is possible that additional focus groups would have uncovered additional themes and concepts. However, we think that this is unlikely because the themes observed in both focus groups were similar. Participants were identified through a single police department, which may not be reflective or representative of other regions. Although participants brought some of these details up on their own, our study did not distinguish between those who observed the violent act(s) directly and those who did not. It may be important in future research to distinguish between the type of exposure a person, child, or family had to the violence to tailor supports appropriately.

## Data Availability

The original contributions presented in the study are included in the article/supplementary material, further inquiries can be directed to the corresponding author.
