# Supplementary material for: Feasibility study exploring sleep impacts among families in the aftermath of firearm-related trauma
Source: Front Sleep. 2026 Aug 4;5:1787037. doi: 10.3389/frsle.2026.1787037 (PMC13481292; doi:10.3389/frsle.2026.1787037)
Supplement: Supplementary file 1 [file Supplementary_file_1.docx]

Interview Protocol

Hello! We are researchers on the faculty at the Brigham and Women’s Hospital and Mass General Hospital interested in learning more about the impact of gun violence in communities. This project is focused on uncovering some of the impacts of gun violence that are not always talked about, like the impact of gun violence on the sleep of families (including surviving caregivers, children, and adolescents). When gun shots go off, especially at night, it can be disturbing. This exposure may make it difficult to fall asleep. When the gun violence affects you personally (e.g., a family member or loved one is involved in the violence) the impacts can be particularly concerning. Our study is designed to address these issues. Today, we will ask you to participate in a brief interview where we talk about these issues of gun violence, community impact, and the impact of gun violence your family may have experienced. There are no right or wrong answers in our conversation today. If anything makes you uncomfortable, it is completely OK to not answer. Do you have any questions before we begin?

**General intro questions**

First, a few questions to get to know you better:

-How old are you?

-How many children do you have/care for?

-What race/ethnicity do you identify as?

-What is your gender?

-Where do you live?

**Sleep and the family**

Now, I am curious to learn a bit more about your sleep and your family’s sleep.

-Could you describe your sleeping arrangement at home?

-Now, how would you describe your sleep overall?

-Next, we are curious to learn about sleep for the child/youth for whom you provide care. Could you tell me about their sleep? Are they generally a ‘good sleeper’?

-How many hours of sleep do you and your child/youth typically get?

-Do you ever notice any issues with your child/youth being sleep during the day?

**Feedback on Moshi**

Next, we are curious to hear your perspective on an intervention we are developing to support families in the wake of gun violence, specifically, supporting those struggling with sleep.

-We are thinking about developing resources that feature information about sleep strategies and techniques like meditation. Do you think that would be helpful?

-Could you tell me a trusted source you would like to receive materials from (e.g., Pediatrician? School counselor? Community organization?)

**Feedback on Moshi**

Now, we want to tell you a bit about the intervention we are thinking about developing to support communities, especially children/youth and their families, that are impacted by gun violence. We are thinking about partnering with a company that creates stories about whimsical characters. These characters have superpowers, such as the ability to quiet their mind. You can program the stories into a child-safe padded portable speaker that a child could keep in their bedroom and listen to before bedtime, or anytime they felt stressed. We are excited to share a few stories with you and get your feedback. Is that ok? [Get approval, proceed]

[Play Moshi story, such as the following example - [https://www.youtube.com/watch?v=3zCXKQ4ggrA](https://www.youtube.com/watch?v=3zCXKQ4ggrA--)]

-What do you like about this story?

-What do you not like about this story?

-Do you think a story/resource like this would be useful to you and your family?

-How frequently might you use something like this?

**How to deliver Moshi**

We would really like your feedback now on how to deliver Moshi. The app can deliver stories either on the app or an external speaker.

-What do you think about listening to the stories on a smartphone? Would that be feasible for your family (see picture below for what a screenshot of Moshi looks like)?

-What do you think about the [project the picture of the speaker] using a speaker like that shown here?

-Do you think this is a feasible idea? What do you think about giving these to families and children impacted by gun violence?

Do you have any other feedback for us on the stories or any other ideas on how we can best support families impacted by these issues?
